# Standardization of cytokine flow cytometry assays

**DOI:** 10.1186/1471-2172-6-13

**Published:** 2005-06-24

**Authors:** Holden T Maecker, Aline Rinfret, Patricia D'Souza, Janice Darden, Eva Roig, Claire Landry, Peter Hayes, Josephine Birungi, Omu Anzala, Miguel Garcia, Alexandre Harari, Ian Frank, Ruth Baydo, Megan Baker, Jennifer Holbrook, Janet Ottinger, Laurie Lamoreaux, C Lorrie Epling, Elizabeth Sinclair, Maria A Suni, Kara Punt, Sandra Calarota, Sophia El-Bahi, Gailet Alter, Hazel Maila, Ellen Kuta, Josephine Cox, Clive Gray, Marcus Altfeld, Nolwenn Nougarede, Jean Boyer, Lynda Tussey, Timothy Tobery, Barry Bredt, Mario Roederer, Richard Koup, Vernon C Maino, Kent Weinhold, Giuseppe Pantaleo, Jill Gilmour, Helen Horton, Rafick P Sekaly

**Affiliations:** 1 BD Biosciences, San Jose, USA; 2 Université de Montreal and CANVAC, the Canadian Network for Vaccines and Immunotherapeutics, Montreal, Canada; 3 National Institute of Allergy and Infectious Diseases, National Institutes of Health, Bethesda, USA; 4 Chelsea and Westminster Hospital and IAVI, London, UK; 5 Uganda Virus Research Institute and IAVI, Entebbe, Uganda; 6 Kenya AIDS Vaccine Initiative (KAVI), University of Nairobi, Kenya; 7 Centre Hospitalier Universitaire Vaudois and EUROVAC, Lausanne, Switzerland; 8 University of Washington and HVTN, Fred Hutchinson Cancer Research Center, Seattle, USA; 9 Duke University Medical Center and HVTN, Durham, USA; 10 Vaccine Research Center, National Institutes of Health, Bethesda, USA; 11 University of California, San Francisco, USA; 12 Merck and Co., West Point, USA; 13 University of Pennsylvania, Philadelphia, USA; 14 Sanofi Pasteur, Lyon, France; 15 Massachusetts General Hospital, Boston, USA; 16 National Institute for Communicable Diseases, Johannesburg, South Africa; 17 Henry Jackson Foundation, Rockville, USA

## Abstract

**Background:**

Cytokine flow cytometry (CFC) or intracellular cytokine staining (ICS) can quantitate antigen-specific T cell responses in settings such as experimental vaccination. Standardization of ICS among laboratories performing vaccine studies would provide a common platform by which to compare the immunogenicity of different vaccine candidates across multiple international organizations conducting clinical trials. As such, a study was carried out among several laboratories involved in HIV clinical trials, to define the inter-lab precision of ICS using various sample types, and using a common protocol for each experiment (see additional files online).

**Results:**

Three sample types (activated, fixed, and frozen whole blood; fresh whole blood; and cryopreserved PBMC) were shipped to various sites, where ICS assays using cytomegalovirus (CMV) pp65 peptide mix or control antigens were performed in parallel in 96-well plates. For one experiment, antigens and antibody cocktails were lyophilised into 96-well plates to simplify and standardize the assay setup. Results (CD4^+^cytokine^+ ^cells and CD8^+^cytokine^+ ^cells) were determined by each site. Raw data were also sent to a central site for batch analysis with a dynamic gating template.

Mean inter-laboratory coefficient of variation (C.V.) ranged from 17–44% depending upon the sample type and analysis method. Cryopreserved peripheral blood mononuclear cells (PBMC) yielded lower inter-lab C.V.'s than whole blood. Centralized analysis (using a dynamic gating template) reduced the inter-lab C.V. by 5–20%, depending upon the experiment. The inter-lab C.V. was lowest (18–24%) for samples with a mean of >0.5% IFNγ + T cells, and highest (57–82%) for samples with a mean of <0.1% IFNγ + cells.

**Conclusion:**

ICS assays can be performed by multiple laboratories using a common protocol with good inter-laboratory precision, which improves as the frequency of responding cells increases. Cryopreserved PBMC may yield slightly more consistent results than shipped whole blood. Analysis, particularly gating, is a significant source of variability, and can be reduced by centralized analysis and/or use of a standardized dynamic gating template. Use of pre-aliquoted lyophilized reagents for stimulation and staining can provide further standardization to these assays.

## Background

Enzyme-linked immunospot (ELISPOT) and cytokine flow cytometry (CFC) (or more specifically, intracellular cytokine staining (ICS)) are popular methods for single-cell analysis of antigen-specific T cell cytokine production. T cell production of IFNγ, and increasingly also IL-2, is taken as a measure of vaccine immunogenicity in experimental vaccine trials. Of the two types of assays, ICS has the advantage of a highly multiparametric read-out (flow cytometry) that allows for precise phenotyping of the responding T cell populations. It has also recently been adapted to a 96-well plate configuration [1,2], allowing for higher throughput analysis similar to that used for ELISPOT. However, while the precision of ELISPOT assays across sites has been recently documented [3], similar studies for ICS assays have been lacking.

Numerous phase I and phase II clinical trials have been initiated using candidate prophylactic HIV vaccines (reviewed in [4]). Many of these trials use ICS as part of their immune monitoring. While most current HIV trials are not powered to determine efficacy, and cytokine production has not been validated as a surrogate marker of protection from HIV infection or progression, there is nevertheless a desire to measure immunogenicity of candidate vaccines as well as safety in early clinical trials [5]. Because many different groups are performing immune monitoring for these clinical trials, there is currently a lack of standardization that would allow accurate comparisons of immunogenicity across candidate vaccines in different clinical trials.

There is some published literature on the intra-and inter-assay precision of ICS assays in whole blood [6]. These values were determined to be about 8% and 20% C.V., respectively. Guidelines for performance of ICS assays have also been recently published [7]. However, there are no existing data documenting the precision of ICS between laboratories, or comparing the precision of ICS using different sample types (e.g., whole blood versus cryopreserved PBMC). In order to allow more meaningful comparisons between laboratories and prioritization of emerging vaccine candidates, and thereby accelerate HIV vaccine development, this ICS standardization study was undertaken.

**Figure 1:**
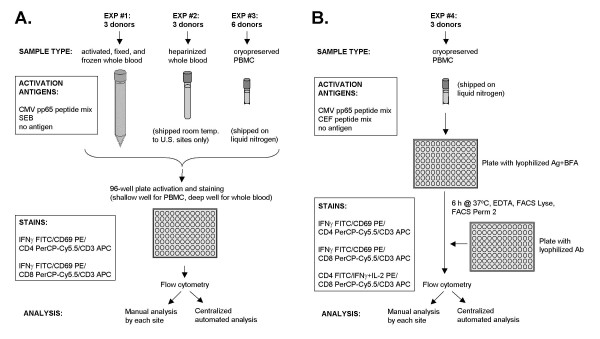
**Experimental design. **(A) Schematic of protocol for Experiments 1–3, performed using liquid antigens and antibodies. (B) Schematic of protocol for Experiment 4, performed using lyophilised antigen and antibody plates.

**Figure 2:**
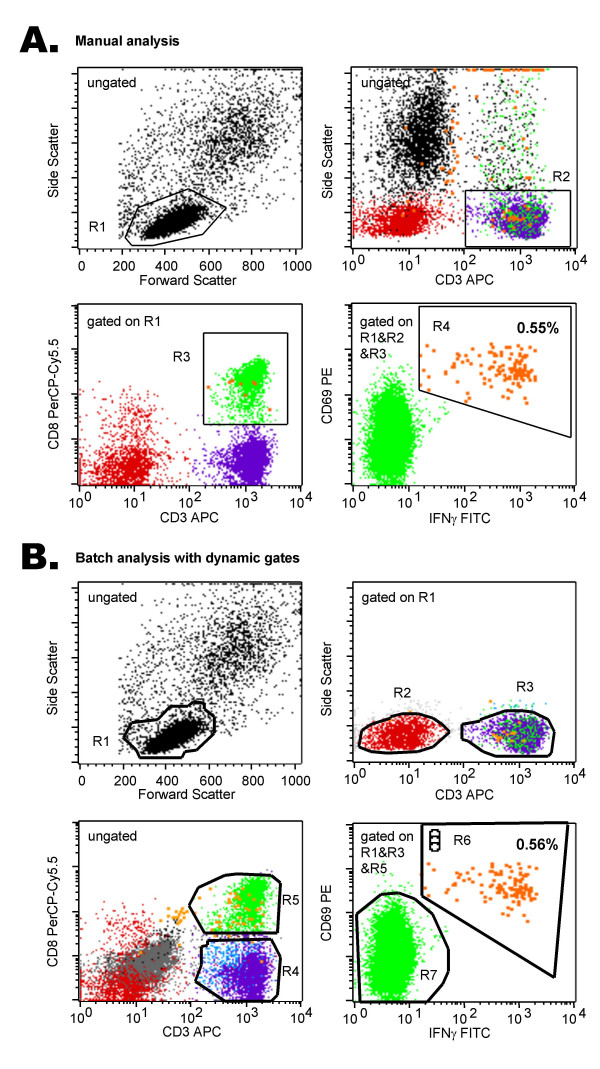
**Manual versus automated gating templates. **(A) Representative manual analysis of a CEF-stimulated sample from Experiment 4. Sequential gates on small lymphocytes, CD3^+ ^cells, and CD3^+^CD8^+^. cells are applied and the percent CD69^+^IFNg^+ ^cells are determined from a plot gated on all of these regions. (B) Dynamic gating template for the same data file as above. Sequential dynamic gates ("Snap-To" gates) are applied as above, except that negative populations are also gated so as to provide a boundary for the movement of the positive region. The percent CD69^+^IFNg^+ ^cells obtained is very similar to that obtained by manual gating in this example, since manual gating was performed so as to include CD3^dim ^and CD8^dim ^cells.

The objectives of the study were three-fold: (1) to assess the reproducibility of ICS assays using different sample types (shipped whole blood vs. cryopreserved PBMC); (2) to determine the inter-laboratory precision of ICS assays among major HIV vaccine clinical research laboratories; and (3) to improve the concordance of methodologies used in these laboratories. To achieve these objectives, joint experiments (Figure 1) were devised using (1) whole blood activated at a central site, then fixed, frozen and shipped to participating laboratories for processing and analysis; (2) fresh whole blood drawn at a central site and shipped to participating labs for activation, processing, and analysis; and (3) cryopreserved PBMC shipped from a central site to participating labs for activation, processing, and analysis. In the latter case, this experiment was also repeated with a larger number of participating laboratories, using pre-formatted microtiter plates containing lyophilised stimuli and lyophilised staining antibodies. In each experiment, raw data files were also sent by the participating labs to a central site for analysis, which was done using a dynamic gating template and batch analysis [1] (Figure 2).

## Results

### Activated, fixed, and frozen whole blood

**Figure 3:**
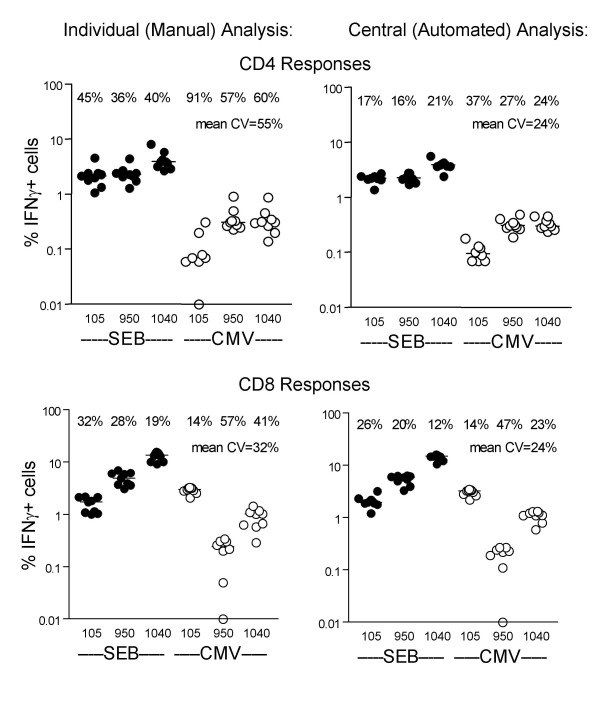
**Results of Experiment 1 (fixed activated whole blood). **IFNγ-positive cells in response to SEB or CMV pp65 peptide mix are expressed as a percentage of CD4^+ ^or CD8^+ ^T cells. Results from each site are indicated as a circle, with median responses for each sample (105, 950, and 1040) indicated by a horizontal bar. The C.V. for each sample is listed across the top of each panel, along with the mean C.V. for that set of samples.

In the first experiment, whole blood from three cytomegalovirus (CMV)-seropositive donors was activated, fixed, and frozen by the method described in Nomura et al.[6]. The blood was incubated for 6 hours in the presence of brefeldin A, either with no stimulus, Staphylococcal enterotoxin B (SEB), or a mixture of overlapping peptides corresponding to the CMV pp65 protein [8-10]. Aliquots of the frozen activated whole blood were then shipped to 9 laboratories for processing and analysis. The results, as reported by each site and also as determined by central, automated analysis of the raw data files, are summarized in Figure 3. For data reported by each site, the mean inter-lab C.V. was 55% for CD4 T cell responses and 32% for CD8 T cell responses. This is higher than the inter-assay C.V. previously reported for ICS assays performed at a single site [6]. However, when the raw data was centrally analyzed, the inter-lab C.V. was reduced to 24% for both CD4 and CD8 T cell responses, very similar to the inter-assay C.V. previously reported [6]. Thus, a large proportion of the site-to-site variability could be explained by differences in gating of the ICS data.

### Fresh whole blood

**Figure 4:**
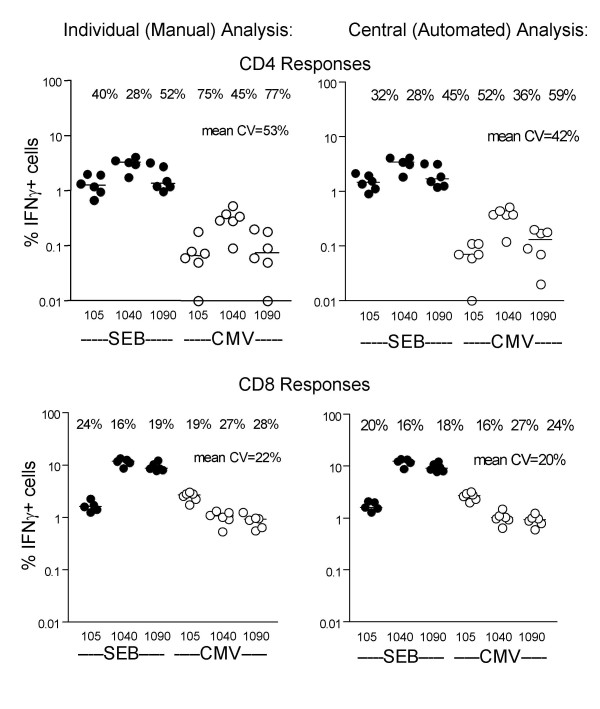
**Results of Experiment 2 (shipped whole blood). **IFNγ-positive cells in response to SEB or CMV pp65 peptide mix are expressed as a percentage of CD4^+ ^or CD8^+ ^T cells. Results from each site are indicated as a circle, with median responses for each sample (105, 1040, and 1090) indicated by a horizontal bar. The C.V. for each sample is listed across the top of each panel, along with the mean C.V. for that set of samples.

In a second experiment, whole blood from three CMV-seropositive donors was shipped overnight to 6 U.S. labs for activation, processing, and analysis. This experiment was conducted twice, since the first trial was compromised by shipping delays. The results of the second trial are shown in Figure 4. As in Figure 3, the inter-lab C.V.'s were higher for data reported by each site, although the reduction due to centralized analysis was less dramatic than in the first experiment. Feedback on gating differences was provided to the labs between the first and second experiment, so the smaller effect of centralized analysis could be attributed to a progression of the individual sites toward a more uniform gating scheme. Also, the relatively high C.V. for CD4 T cell responses (42% even after centralized analysis) could be due to the low mean response to CMV peptide mix in two of the donors (donors were not identical in the different experiments). Since the C.V. varies inversely with the mean of the sample population, comparison of C.V.'s between experiments performed on different donors are subject to this confounding variable.

### Cryopreserved PBMC

**Figure 5:**
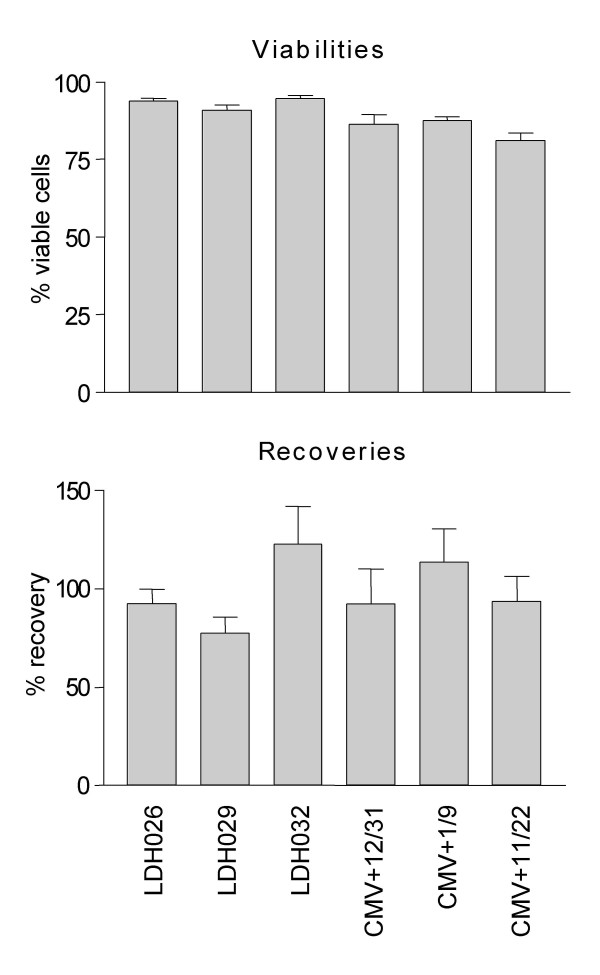
**Viabilities and recoveries for cryopreserved PBMC samples used in Experiment 3. **Error bars represent SEM of the 6 sites participating.

In a third experiment, PBMC were isolated from 6 CMV-seropositive donors and cryopreserved. Replicate cryopreserved vials were sent to each of 7 sites, where they were thawed, rested overnight, stimulated, processed, and analyzed. The post-thaw viability and recovery of the PBMC samples from each site are shown in Figure 5. Mean viabilities were >82%, and mean recoveries were >75% for each sample (determined by trypan blue exclusion). In general, viabilities were quite consistent across labs, while recoveries varied more widely, both between labs and between samples. This could be due in part to imprecise filling of the vials when they were initially frozen, which would impact the apparent recoveries calculated upon thawing. Furthermore, while a common thawing protocol was provided, no attempt was made to standardize counting methods or other factors that may impact the reproducibility of viability and recovery calculations across labs.

**Figure 6:**
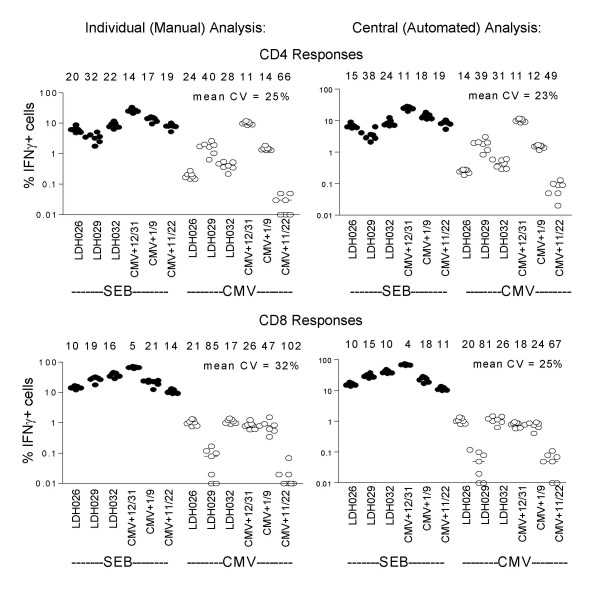
**Results of Experiment 3 (cryopreserved PBMC). **IFNγ-positive cells in response to SEB or CMV pp65 peptide mix are expressed as a percentage of CD4^+ ^or CD8^+ ^T cells. Results from each site are indicated as a circle, with median responses for each sample indicated by a horizontal bar. Sample names are listed along the X axis. The C.V. for each sample is listed across the top of each panel, along with the mean C.V. for that set of samples.

The ICS results from cryopreserved PBMC are shown in Figure 6. The inter-lab C.V.'s for this experiment averaged slightly lower than those for the whole blood experiments (25–32% for manual analysis; 23–25% for centralized automated analysis). Like the fresh whole blood experiment, the improvement in C.V. from centralized analysis was relatively small. When outlier samples with unusually low responses were checked for viability and recovery, they were not necessarily low in these parameters as well. In fact, there was no obvious relationship of viability or recovery with response, perhaps because viabilities and recoveries were virtually all within generally acknowledged limits of acceptability (>80% viability, >50% recovery) [11,12].

### Cryopreserved PBMC with preconfigured lyophilised reagent plates

To expand upon the results of the third experiment using cryopreserved PBMC, a fourth experiment with this sample type was carried out using an enlarged cohort of participating laboratories (table 1). In addition, a protocol refinement was introduced to attempt to further reduce inter-lab variability: Peptide stimuli together with brefeldin A were provided in lyophilised form in appropriate wells of a microtiter plate, to provide simplified assay set-up; and lyophilised staining antibody cocktails were provided in the corresponding wells of a second microtiter plate. These latter were rehydrated and added to the cells in the first microtiter plate after fixation and permeabilization of the cells. This experiment also sought to compare two different types of staining antibody cocktails: the two cocktails used in the previous three experiments (IFNγ FITC/CD69 PE/CD4 PerCP-Cy5.5/CD3 APC and IFNγ FITC/CD69 PE/CD8 PerCP-Cy5.5/CD3); and one that combined CD4 and CD8 staining in a single sample, as well as adding IL-2 staining (CD4 FITC/IFNγ +IL-2 PE/CD8 PerCP-Cy5.5/CD3 APC).

**Figure 7:**
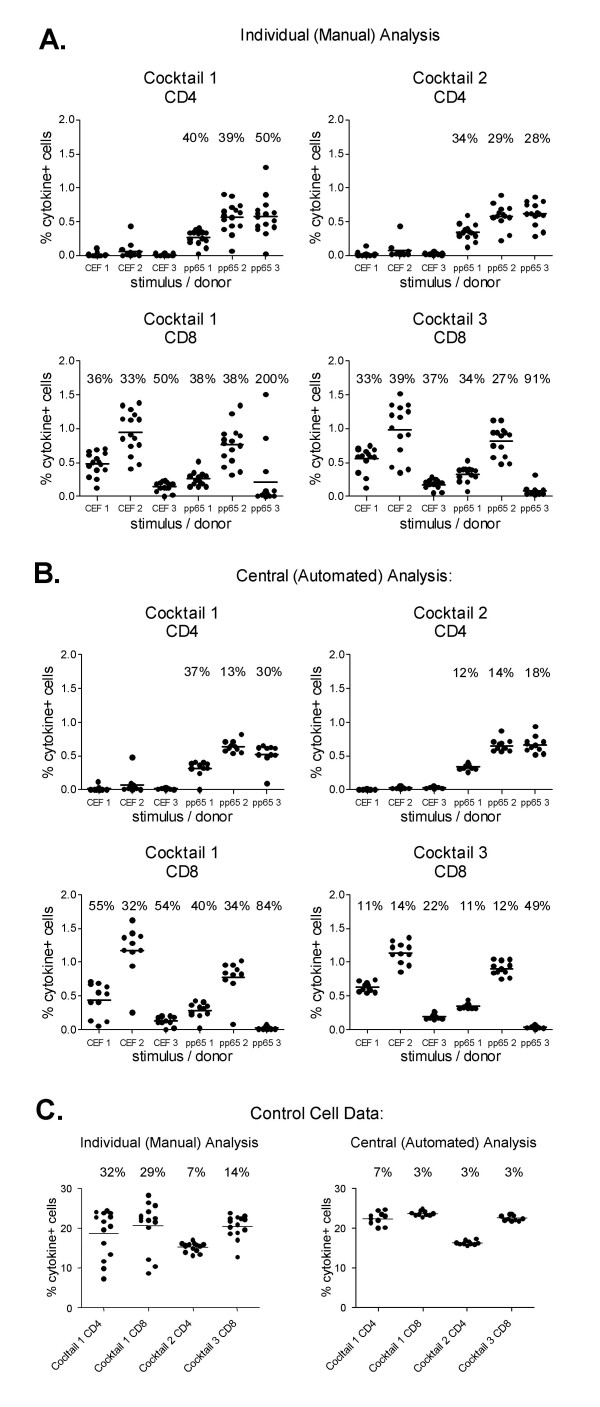
**Results of Experiment 4 (cryopreserved PBMC with lyophilized reagents). **Cytokine-positive cells in response to CEF peptides or CMV pp65 peptide mix are expressed as a percentage of CD4^+ ^or CD8^+ ^T cells. Results from each site are indicated as a circle, with median responses for each sample indicated as a horizontal bar. Sample names are listed along the X axis. The C.V. for each positive sample is listed across the top of each panel, along with the mean C.V. for that set of samples. (A) Data reported by individual sites. (B) Data after centralized analysis using a dynamic gating template. Cocktail 1 consisted of CD4 FITC/IFNγ +IL-2 PE/CD8 PerCP-Cy5.5/CD3 APC. Cocktail 2 consisted of IFNγ FITC/CD69 PE/CD4 PerCP-Cy5.5/CD3 APC, and cocktail 3 consisted of IFNγ FITC/CD69 PE/CD8 PerCP-Cy5.5/CD3 APC. (C) Control cell data from Experiment 4. Activated, processed, and stained PBMC were lyophilised and run as controls by each site in Experiment 4. These cells had been stained with cocktail 1, 2, or 3. The left panel shows data reported by individual sites; the right panel, data after centralized analysis using a dynamic gating template. Most of the inter-lab variability was removed by this method of analysis.

The results of this experiment are shown in Figure 7 (note the change to a linear scale in this and the following figures). A set of peptides consisting of epitopes from CMV, EBV, and influenza (CEF) [13] was used as a positive control, due to restrictions on international shipment of SEB. CEF was expected to induce CD8, rather than CD4 responses, and indeed the CD4 responses to this control were very low or negative. The CD8 responses, while positive, were considerably lower than those seen with SEB in the previous experiments. Responses to CMV pp65 peptide mix were also not high in the donors used in this experiment, but were detectable in both CD4 and CD8 compartments. Despite the lower response means, the average C.V. for this experiment was roughly similar to the previous experiment, when comparing the same staining antibody cocktails (cocktails 2 and 3). For unknown reasons, the average C.V. for cocktail 1 (CD4/IFNγ +IL-2/CD8/CD3) was higher than that for cocktails 2 and 3, although the mean percentage of cytokine-positive cells was not significantly different. The addition of IL-2 in this cocktail did not significantly increase the mean percentage of cytokine-positive cells, as very few cells responding to CMV produce IL-2 without IFNγ [14]. This would not be expected to be true for all types of responses, however.

When data for this experiment were centrally analysed (Figure 7B), the average C.V.'s were considerably reduced, much like in the first experiment (Figure 3). This could reflect the fact that new laboratory sites had been added that had not yet standardized their gating strategies with the existing sites; thus more benefit was realized by centralized analysis. The difference in average C.V. between cocktail 1 and cocktails 2 and 3 was preserved even after centralized analysis. The mean C.V. for cocktails 2 and 3 was now 18%, the lowest variability seen in any of the experiments. For comparison, the mean inter-lab C.V. of the percent CD4^+ ^or CD8^+ ^cells in the unstimulated samples from this experiment was 3% and 7%, respectively (data not shown).

### Lyophilised control cells

As a positive control in Experiment 4, a set of PBMC were SEB-activated, processed, stained, and then lyophilised in certain wells of the lyophilised antibody plates. They were hydrated and transferred to the plate containing activated cells, along with the staining antibodies. These cells served as a control for instrument setup and gating, since all the activation and processing steps were done centrally. The results reported by the individual sites for these cells are shown in the left panel of Figure 7C. Surprisingly, the average C.V. (20.5%) was only slightly lower than that for the rest of Experiment 4, in which cells were activated and processed independently by each site. However, when the control cell data were centrally analysed using a dynamic gating template (right panel), the C.V.'s were reduced to 3–7%. This reinforces the notion that the vast majority of inter-lab variability is due to gating.

### Spontaneous cytokine production in the three sample types

**Figure 8:**
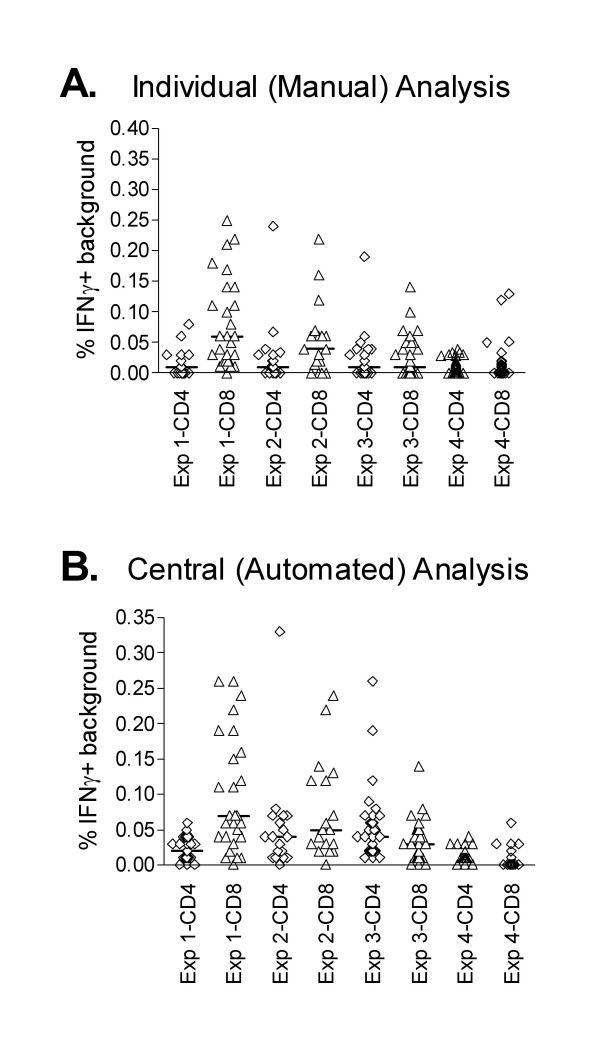
**Background cytokine-producing cells by experiment. **Cytokine-positive cells in the absence of stimulus are expressed as a percentage of CD4^+ ^or CD8^+ ^T cells. Each symbol represents the background from a single sample processed by a single site. Medians are shown by a horizontal bar. Data from experiment 4 are for cocktails 2 and 3 only, to be comparable with experiments 1–3.

"Background" or spontaneous cytokine production (subtracted from all data in Figures 3, 4, 6, and 7) is plotted for all experiments in Figure 8. Backgrounds were generally low. For all experiments combined, the mean CD4 background was 0.02% and the mean CD8 background was 0.05%. 98% of CD4 samples and 84% of CD8 samples had backgrounds =0.1%. There were a significant number of CD8 samples that exhibited high spontaneous cytokine production. However, the mean CD8 background was significantly higher than the mean CD4 background only in the activated, fixed whole blood experiment (p < 0.0005). When centralized automated analysis was applied to the data, backgrounds were not usually reduced. This indicates that the gains in reproducibility seen with centralized analysis were not simply due to reductions in background.

While CD4 backgrounds were very similar between experiments, CD8 backgrounds varied. The median CD8 background in the PBMC experiments was significantly lower than that of the frozen activated whole blood experiment (p < 0.0001) or the fresh whole blood experiment (p < 0.05). The differences were significant after centralized analysis as well. However, this could be due to the fact that different donors were used in the four experiments, rather than being due to any inherent difference between assay types. In experiment 4, the CD4 backgrounds for cocktail 1 were significantly higher than those for cocktail 2 (p < 0.05, data not shown), while there was no significant difference for CD8 backgrounds. This could be due to the inclusion of IL-2 in cocktail 1, which would be expected to be produced by more CD4^+ ^than CD8^+ ^cells, and thus contribute selectively to the CD4 background.

## Discussion

This study examined the reproducibility of ICS assays across sites using different assay formats. It was not designed to compare ICS with other immune monitoring assays, comparisons of which have been published [15-21]. The current study used 96-well plate-based protocols exclusively, as these were considered more convenient, and have recently been validated against tube-based protocols for both PBMC and whole blood [1].

**Figure 9:**
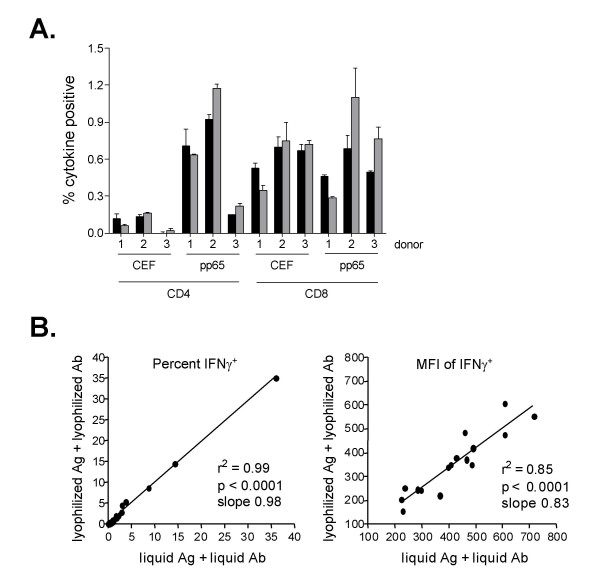
**Comparison of liquid and lyophilised reagents. **Comparative results are shown with backgrounds subtracted; no significant differences in backgrounds were seen with liquid versus lyophilised reagents (data not shown). (A) Data from one site that compared cocktail 1 (CD4 FITC/IFNγ +IL-2 PE/CD8 PerCP-Cy5.5/CD3 APC) in liquid and lyophilised form in Experiment 4. Black bars indicate liquid antibodies, grey bars indicate lyophilised antibodies. Error bars indicate SEM of duplicate wells. (B) Combined comparison of liquid antigen + liquid antibodies versus lyophilised antigen + lyophilised antibodies. Whole blood was activated with either SEB or pp65 peptide mix, and the percentage of IFNγ^+ ^cells (CD4^+ ^or CD8^+^) were compared with liquid versus lyophilised reagents (left panel). A similar comparison was made for the mean fluorescence intensity (MFI) of IFNγ^+ ^cells (CD4^+ ^or CD8^+^) (right panel). Similar results were obtained using PBMC (not shown).

Lyophilised reagents in plates were used for Experiment 4. These have been extensively compared to liquid reagents ([22] and Figure 9) and shown to be largely equivalent. In addition to convenience of assay set-up, the lyophilised reagent plates offer long reagent stability, even at room temperature (>1 year, data not shown), and a potential reduction in errors caused by incorrect reagent addition. Intra-plate variability using lyophilised reagents was determined to be <10% in ICS assays (data not shown).

There are some potential drawbacks to the use of 96-well plates. One of these is the possibility of well-to-well contamination during the assay. This was observed in an initial subset of Experiment 4 (data not shown), in which some sites received lyophilised plates with SEB as a positive control. Some of these sites experienced high backgrounds in the negative control wells adjacent to the SEB-containing wells. It was later determined that cross-contamination probably occurred during the initial distribution of the antigens on the plates, and this was compounded by the fact that the donors used were unusually sensitive to SEB stimulation (responses >30% of CD4^+ ^and CD8^+ ^T cells). When SEB was replaced with CEF as a positive control, no such problems were noted. This experience suggests that the choice and placement of positive control wells on a plate deserves consideration.

The current study was designed to determine inter-lab variability in ICS assays. As such, there were no data "filters" applied to exclude potentially erroneous data or outliers. However, improved precision of ICS results might be obtained if certain acceptance criteria were applied before data were taken as valid. For example, a minimum number of collected events could be specified (sites in this study were asked to collect 10,000–40,000 CD4^+ ^or CD8^+ ^T cells per sample, or 60,000 CD3^+ ^cells). This number of events was designed to yield precision levels that would minimize event number as a factor in inter-lab reproducibility. There could also be acceptance criteria based upon the absolute level of background, or the degree of reproducibility between duplicate samples, if run (the current study did not use duplicate samples).

It is also possible to apply statistics to derive further meaning from numerical results. For example, statistical tests could be used to determine whether a given response can be discriminated from a given background, for a particular number of events collected [23,24]. This can be given by a power calculation as follows:

N = [2*P_av_(1-P_av_)(Z_α _+Z_β_)^2^]/Δ^2^

where N is the number of events in each sample needed for significance, P_av _is the average proportion (between the background and test samples), and Δ is the difference between these two proportions. The term (Z_α _+Z_β_)^2 ^is referred to as a power index, and varies depending upon the desired power and p value. For example, (Z_α _+Z_β_)^2 ^= 23.9 for 99% power and p < 0.005 [23].

In addition, a confidence interval could be derived around the difference of the test result and the negative control [24], in order to allow discrimination of significant differences between various samples. Other statistical methods have also been employed in order to determine cut-off values for positive responses in ICS [25,26]. No attempt was made in the current study to define which results were positive, as all data were reported objectively, and all donors were known to be CMV seropositive.

Examination of the data from Figures 3, 4, 6, and 7 suggests that samples with a low number of cytokine-positive cells had higher variability than samples with a high number of cytokine-positive cells. The relationship of response level and C.V. is summarized in Table 2 for all assays (CD4 and CD8, whole blood and PBMC) considered together. These data emphasize the difficulty of achieving precise results at response levels of less than 0.1% of CD4 or CD8 T cells. For these samples, collecting even more events than what was suggested would be expected to improve precision, per the discussion above.

The average C.V. across the four experiments is summarized in Table 3. These data are confounded by the fact that different donors and different laboratories participated in the four experiments. However, variability due to individual analysis can be excluded by comparing only centrally analysed data (bottom row of Table 3). Assuming no effect from the other confounding variables, we found that Experiment 4 (cryopreserved PBMC with lyoplates) yielded a significantly lower average C.V. than Experiment 2 (shipped whole blood) (p < 0.05). Also, the average C.V. of centrally analysed data from all experiments was significantly lower than that of individually analysed data (p < 0.0001). This highlights the amount of variability in each experiment that is due to gating differences between sites.

Mitigation of gating variability was achieved in these experiments by centralized analysis with a dynamic gating template (see Figure 2B). The dynamic gating template allowed for more automated, batch analysis of the data. Once such a template was created and optimized (see Materials and Methods section for description), it could also have been provided to individual sites in order to yield the same results. It is further possible that similar results could be achieved by manual analysis, provided it was done by a single operator. Standardization of gating techniques, in the absence of centralized analysis or dynamic gating templates, could also improve precision. The improvement in C.V. made by centralized analysis was most marked in the first experiment, and progressively less in experiments 2 and 3, perhaps because of standardization of gating among sites over time. Experiment 4 included many new sites, and the improvement in C.V. from centralized analysis was again more marked.

**Figure 10:**
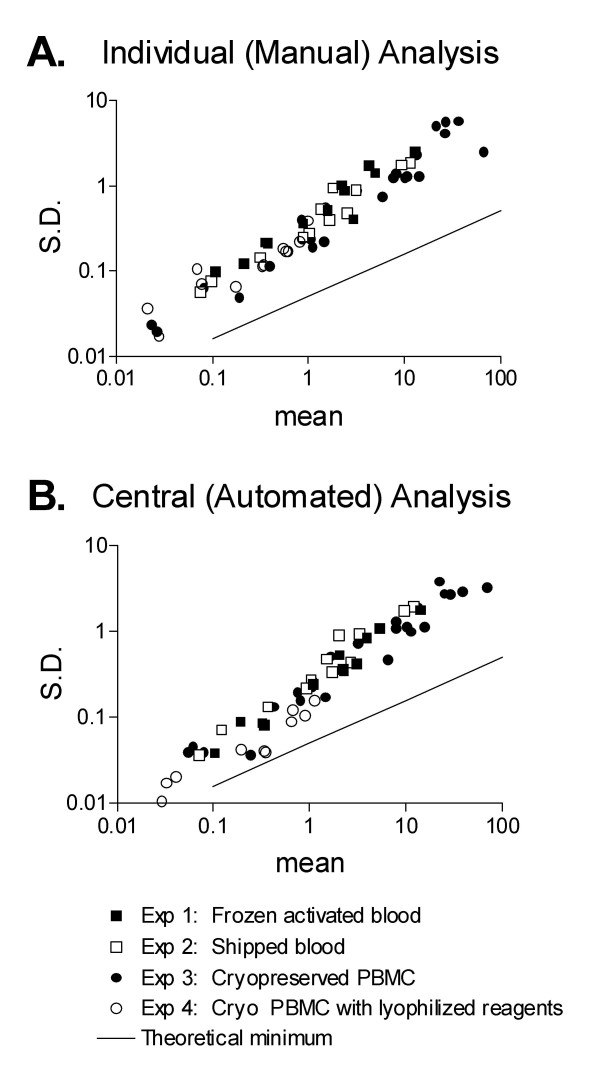
**S.D. versus mean for all assays. **A linear relationship between S.D. and mean is expected based upon counting statistics [23]. This expected relationship (for a data set of 40,000 events) is shown by the solid black line. The actual data from the four experiments is shown in the symbols. Data from experiment 4 are for cocktails 2 and 3 only, to be comparable with experiments 1–3. The difference (in the Y dimension) between the data points and the solid line represents variability from sources other than counting statistics. Note that the data points for all three assay types cluster together, indicating that variability is similar for all three assay types. When individual analysis variability is removed (B), there is a slight tendency toward lower variability with cryopreserved PBMC (solid circles), and higher variability with shipped whole blood (open squares). The tendency toward lower variability is more pronounced in the experiment using cryopreserved PBMC with lyophilised reagents (panel B, open circles).

Because the C.V. varies as a function of the response level (Table 2), it is possible that differences in mean C.V. between assay formats were due to the number of low versus high responders in each experiment (since different donors were used in the four experiments). Also, the C.V. is highly sensitive to small changes in the mean, when the mean is a very low number. Therefore, an analysis of S.D. versus mean was also performed for the four experiments (Figure 10). This data confirms the data of Table 3, indicating that the three assay formats showed grossly similar reproducibility. However, when analysis variability was removed, cryopreserved PBMC assays appeared to be slightly more reproducible than shipped whole blood assays. This seemed especially apparent in experiment 4, where lyophilised reagents were used.

In addition to differences in reproducibility, the various assay formats have other benefits and drawbacks as well. Cryopreserved PBMC are much more amenable to peptide (and superantigen) stimulation than to whole protein stimulation [9]; while whole blood assays are equally amenable to stimulation with either type of antigen. Also, consistently good cryopreservation of PBMC at multiple clinical sites is difficult to achieve, but highly important for achieving reproducible results with PBMC [27,28] (DeLaRosa et al., manuscript in preparation). This could become less of a factor if a stabilizing matrix for preserving whole blood or PBMC function during shipping were discovered. All in all, the choice of assay format for a clinical trial will depend not only upon considerations of assay precision, but also upon the type of antigen(s) used and the capabilities of the participating clinical sites.

The use of lyophilised reagent plates appeared to reduce inter-lab variability. This conclusion cannot be drawn with certainty, because different participating laboratories and different donors were used between experiments 3 and 4. However, it is intriguing to note that, when centrally analysed data was compared (to remove gating as a source of variability), the mean C.V.'s of experiment 4 were the lowest of all four experiments (18%, Table 3). This is despite the fact that the donors and stimuli used in experiment 4 resulted in lower mean response levels, which should tend to increase the C.V. This is also borne out by the analysis of Figure 11B, where the results for experiment 4 appeared to be generally closer to the theoretical minimum SD than did the results for the other experiments.

With the possibility of achieving inter-laboratory C.V.'s of less than 20%, even with relatively low responses, ICS compares favourably to ELISPOT, for which interassay C.V.'s of 17–18% for PHA and 55–65% for Candida have been reported [29,30]. ICS is also comparable to cytokine ELISA, the latter having reported interassay C.V.'s of <25% [31,32]. Phenotypic staining, such as used for CD4 counting, can achieve higher precision levels than functional assays, and averaged around 10% C.V. in one multisite study [33]. For comparison, the inter-lab C.V. of the CD4^+ ^or CD8^+ ^cell percentages was around 5% in experiment 4 of the present study (data not shown). CD4 counting precision has also been shown to be dependent upon the number of events collected, gating, and use of automated analysis [33,34]. Since functional assays are subject to more variables than phenotypic staining, the ability to achieve precision levels such as those reported here should be considered favourable. ICS could thus be a viable tool for comparing immune responses even across clinical trials, provided the methodology was standardized.

## Conclusion

ICS assays could be performed with inter-laboratory C.V.'s of approximately 20% at response levels of >0.5%, and C.V.'s of approximately 25–30% at response levels of 0.1–0.5%. The C.V. increased further at response levels of =0.1%. A significant portion of inter-laboratory variability could be eliminated by use of centralized analysis and/or a dynamic gating template.

Whole blood and cryopreserved PBMC showed grossly similar levels of reproducibility. However, when analysis variability was removed, cryopreserved PBMC processed with lyophilized reagents showed significantly better reproducibility than shipped whole blood. Shipped whole blood assays were also subject to data loss when samples were not delivered in a timely fashion.

Background cytokine production was mostly =0.05% for both CD4 and CD8 cells. While CD8 backgrounds were lower in cryopreserved PBMC than in whole blood, this could have been due to the use of different donors in the four experiments. With the high viabilities and recoveries obtained for cryopreserved PBMC in this study, there was no obvious relationship between viability/recovery and response.

The use of microtiter plates containing lyophilised reagents simplified the ICS protocol, and appeared to improve assay reproducibility. This format lends itself to international shipping of reagents (because there is no need for refrigeration), and also to larger clinical trials (because of the stability of the lyophilised reagents). It is also a way to reduce the chance of pipetting errors, because the plates are pre-formatted.

The results of this study indicate that ICS assays can be reasonably standardized between sites, but that considerations of sample format and expected response levels can influence the precision of the results. These data should guide comparisons of ICS results between different groups or in different clinical trials.

## Methods

### Whole blood preparation

Heparinized whole blood was collected from healthy CMV seropositive volunteers for experiments 1 and 2. For experiment 1, the blood was activated in 15 mL conical tubes according to the method of Nomura et al.[6]. Activated blood was treated with 2 mM final concentration of EDTA for 15 minutes at room temperature, then 10 volumes of FACS Lysing Solution (BD Biosciences, San Jose, CA) were added. After 10 minutes at room temperature, the tubes were frozen at -80°C, then shipped to participating laboratories on dry ice. The protocol used by each laboratory for handling these samples is provided in Additional File 1.

For experiment 2, 5 mL of heparinized whole blood was overnight shipped in an insulated container at ambient temperature to each participating lab. The protocol used by each lab for handling these samples is provided in Additional File 2.

### PBMC preparation and cryopreservation

For experiments 3 and 4, PBMC from leukapheresis of CMV seropositive donors were isolated using Ficoll gradient separation. They were then cryopreserved according to a standard protocol (Disis et al., submitted for publication). These cryopreserved PBMC were shipped to participating labs using liquid nitrogen dry shippers. The protocol used by each lab for thawing and processing of these cells is provided as Additional Files 3 and 4.

### Instrumentation and setup

The flow cytometry instrumentation used in this study included 12 BD FACS Caliburs (BD Biosciences), 3 BD LSRIIs (BD Biosciences), and 1 CyAn (Dako Cytomation, Fort Collins, CO). Instrument setup was at the discretion of the individual laboratory, and was either manual (using isotype control stained cells to set PMT voltages, and single-stained cells to set compensation) or automated (using BD FACSComp software and BD Calibrite beads (BD Biosciences)). In some labs, automated setup was followed by manual adjustment using stained cells as above.

### Dynamic gating templates

Original FCS files from each site were sent to BD Biosciences for analysis using a dynamic gating template (Figure 2B). This template was built using "Snap-To Gating" and "Tethering" tools available in CellQuest Pro software (BD Biosciences). The shape of the snap-to gates is determined by a clustering algorithm, and this algorithm allows for their movement from sample to sample in a data-dependent manner. The size and amount of allowable movement of each snap-to gate was adjusted by inspection of a subset of the files to be used, with iterative changes being made until the template performed as desired. The template was then used, without further adjustment, on all the files of a given experiment. Since the template was generated in CellQuest Pro software, only files generated on FACS Calibur instruments were analyzable by this method.

### Statistical analyses

The %CV was calculated as 100*SD/mean for each sample, from the percentage of cytokine-positive cells reported by each laboratory or derived from centralized analysis of that sample. The mean CV for each experiment was taken as the average of all the individual sample CVs. Statistical significance of differences in the average CV between experiments was calculated using a Kruskal-Wallis test, with Dunn's Multiple Comparison test to determine where significant differences were found. The significance of the difference between individually and centrally analyzed data was calculated by comparing the aggregate CVs of all samples from all experiments using a Wilcoxin signed rank test for matched pairs. A two-tailed Student t test was used to calculate significance of differences in background within or between experiments.

## Authors' contributions

HTM compiled the data and drafted the manuscript. AR coordinated the study. P.D'S. and J.D. secured logistic and financial support. All authors helped design the study, supervised and/or carried out the experiments, and provided editorial comments and assistance.

## Supplementary Material

Additional File 1 Protocol for fixed, activated whole blood (Experiment 1) Click here for file

Additional File 2 Protocol for shipped whole blood (Experiment 2) Click here for file

Additional File 3 Protocol for cryopreserved PBMC (Experiment 3) Click here for file

Additional File 4 Protocol for cryopreserved PBMC with lyophilised antigen and antibody plates (Experiment 4) Click here for file

## Figures and Tables

**Table 1:** Study participants and institutions.

Institution (Consortium)	Participants	Participated in:			
		
		Exp. 1 (fixed activated blood)	Exp. 2 (shipped whole blood)	Exp. 3 (cryo-preserved PBMC)	Exp. 4 (cryo-preserved PBMC with lyoplates)
University of Montreal (CANVAC)	Rafick Sekaly, Eva Roig, Claire Landry	x		x	x
Chelsea and Westminster Hospital (IAVI)	Jill Gilmour, Peter Hayes	x		x	x
Uganda Virus Research Institute (IAVI)	Josephine Birungi, Omu Anzala				x
Centre Hospitalier Universitaire Vaudois (EUROVAC)	Giuseppe Pantaleo, Alexandre Harari, Miguel Garcia	x		x	x
Fred Hutchison Cancer Research Center (HVTN)	Helen Horton ,Ruth Baydo, Ian Frank	x	x	x	x
Duke University (HVTN)	Kent Weinhold, Janet Ottinger, Megan Baker, Jennifer Holbrook	x	x		x
Vaccine Research Center, NIH	Mario Roederer, Richard Koup, Laurie Lamoreaux	x	x	x	x
Merck and Co.	Timothy Tobery, Lynda Tussey, Kara Punt	x	x	x	x
University of California, San Francisco	Barry Bredt, Elizabeth Sinclair, Lorrie Epling	x	x		x
BD Biosciences	Vernon Maino, Holden Maecker, Maria Suni	x	x	x	x
Sanofi Pasteur	Nolwenn Nougarede, Sophia El-Bahi				x
National Inst. Communicable Diseases, South Africa	Clive Gray, Hazel Maila				x
Massachusetts General Hospital	Marcus Altfeld, Gailet Alter				x
University of Pennsylvania	Jean Boyer, Sandra Calarota				x
Henry Jackson Foundation	Josephine Cox, Ellen Kuta				x

Financial and Operational Support: Aline Rinfret, CANVAC; Patricia D'Souza, NIAID, NIH; Janice Darden, NIAID, NIH

**Table 2:** Percent C.V. by mean percent cytokine-positive T cells.

Mean % cytokine-positive cells		= 0.1%	0.1 – 0.5%	>0.5%
Number of samples in range		6	6	35

Average percent C.V.	Individual (Manual) Analysis	71%	31%	21%
	Central (Automated) Analysis	56%	25%	18%

**Table 3:** Percent C.V. by assay format.

Assay Type		Fixed Activated Blood	Shipped Whole Blood	Cryopreserved PBMC	Cryopreserved PBMC with lyophilised reagents
Number of samples		12	12	24	9

Average percent C.V.	Individual (Manual) Analysis	44%	38%	28%	39%
	Central (Automated) Analysis	24%	31%	23%	18%
