# Supplementary material for: Standardization of cytokine flow cytometry assays
Source: BMC Immunol. 2005 Jun 24;6:13. doi: 10.1186/1471-2172-6-13 (PMC1184077; doi:10.1186/1471-2172-6-13)
Supplement: Additional File 1 — Protocol for fixed, activated whole blood (Experiment 1) [file 1471-2172-6-13-S1.pdf]

CFC Standardization Protocol: Analysis of Activated, Fixed, and Frozen Whole Blood

Description

This protocol is for permeabilization, staining, and analysis of previously activated, fixed, and frozen whole blood in 96-well deep well plates. The protocol is designed for four-color staining of IFN[illegible]-producing cells using a CD4 and a CD8 T cell staining cocktail.

Materials and Methods

Table 1 CFC Staining Reagents

CFC Reagents	Source	Catalog Number
BD FastImmune CD4 Intracellular IFN[illegible] Detection Kit[b]	BDIS	Supplied
BD FastImmune CD8 Intracellular IFN[illegible] Detection Kit	BDIS	Supplied
Deionized water[c]		
Paraformaldehyde, 10%	Electron Microscopy Sciences	15712-S
PBS 1X[c]		
Bovine serum albumin (BSA)[c]		
NaN_3[c]		
BD CaliBRITE™ FITC + PE beads	BDIS	349502
BD CaliBRITE PerCP-Cy5.5 beads	BDIS	345036
BD CaliBRITE APC beads	BDIS	340487

[a] BDIS: BD Biosciences, Immunocytometry Systems

[b] BD FastImmune intracellular detection kits include Brefeldin A, EDTA, BD FACS Lysing Solution, and BD FACS Permeabilization Solution 2.

[c] No specific manufacturer recommended

Table 2 Accessory Products and Instrumentation

Product	Source	Catalog Number
96-well deep well conical bottom plate	BDDL[a]	353966
Lid for 96-well deep well conical bottom plate	BDDL	351191
Single- and multi-channel pipettors and tips[b]		
Serological pipettor[b] (Pipet-Aid or equivalent) and pipets		
Table top centrifuge with deep well plate holders[b] (e.g. Sorvall RT6000 centrifuge, plate holder catalog #11093)		
BD FACSCalibur brand flow cytometer	BDIS[a]	
BD Multiwell Autosampler (optional)	BDIS	342364
35 mm multiwell plate aspirator manifold	V&P Scientific, Inc. San Diego, CA 92121	VP 187A
Vacuum source for above[b]		

[a] BDIS: BD Biosciences, Immunocytometry Systems. BDDL: BD Biosciences, Discovery Labware

BD Biosciences

[b] No specific manufacturer recommended

Please follow all recommended precautions that are provided in the technical data sheet of each manufacturer's product.

Instructions for Processing Reagents

FACS Permeabilizing Solution 2 from FastImmune kit
Dilute 10X solution in deionized water to make 1X working solution. Store at room temperature.

Paraformaldehyde in PBS, 1%
Dilute 10% solution of paraformaldehyde 1:10 in 1X PBS. Store at 4ºC.

Wash buffer
First prepare stock solutions of 5% BSA in deionized water (filter sterilize) and 10% NaN_3 in deionized water. Then prepare 500 mL of wash buffer by adding 50 mL of 5% BSA stock solution and 5 mL of 10% NaN_3 stock solution to 445 mL of 1X sterile PBS. This represents final concentrations of 0.5% BSA and 0.1% NaN_3 in PBS. Store at 4ºC.

Protocol

Thawing and aliquoting of fixed, activated blood

1. Fixed, activated whole blood should be stored at –80C prior to use.
2. Thaw tubes briefly in a 37C water bath (do not allow blood to warm completely to 37C).
3. Remove tubes promptly, and invert to mix. For unstimulated and peptide mix stimulated tubes, dispense 1.5 mL into each of two wells of a 96-well deep well plate. For SEB stimulated tubes, dispense 1.5 mL into each of four wells (two wells will be used for isotype controls). Extra wells may also be run for additional stains of interest.
4. Centrifuge plate at room temperature at 500 x g for five minutes.

Permeabilization and Staining

1. Aspirate supernatant. Add 1 mL of BD FACS Permeabilizing Solution 2 per well, pipetting up and down to resuspend each pellet. Incubate at room temperature for 10 minutes.
2. Add 0.5 mL of wash buffer per well and centrifuge at room temperature at 500 x g for five minutes.
3. Aspirate supernatant. Add 1.5 mL of wash buffer, pipetting up and down to resuspend each pellet. Centrifuge at room temperature at 500 x g for five minutes.
4. Aspirate supernatant. Add 20 μL of appropriate mAb cocktail to each well. For each stimulation, stain one well with anti-IFNγ/CD69/CD4/CD3 and one well with anti-IFNγ/CD69/CD8/CD3. For SEB stimulations, stain two additional wells with IgG2a/IgG1/CD4/CD3 and IgG2a/IgG1/CD8/CD3, respectively. As each staining cocktail is added, pipet up and down to resuspend pellet in each well. Incubate for 60 minutes at room temperature in the dark.
5. Add 1.5 mL of wash buffer and centrifuge at room temperature at 500 x g for five minutes.
6. Aspirate supernatant. Repeat steps 5 and 6 one additional time.
7. Resuspend pellet with 200 μL cold 1% paraformaldehyde.
8. Keep plate at 4°C in the dark until FACS acquisition, which should be performed within 24 hours.

Acquisition

1. Using BD FACSComp™ software and BD CaliBRITE™ reagents, set up BD FACSCalibur using "Lyse No Wash" settings.
2. Make sure cells are well suspended before acquisition.

3. Acquire on BD Multiwell Autosampler, if available. Set acquisition template to stop after collecting 20,000 CD3+CD4+ lymphocytes, or 20,000 CD3+CD8+ lymphocytes, or after 180 seconds. Store all lymphocytes defined by FSC vs. SSC.
4. Analyze by setting a gate on either CD3+CD4+ lymphocytes, or CD3+CD8+ lymphocytes, and displaying gated plot of anti-IFN□ vs. CD69 for each sample. Using an SEB-stimulated samples, draw a region that encompasses all double-positive cells in this plot. Check that this region does not include cells in the IFN□-negative population of the other samples. Report double-positive cells in this region for each sample.
5. Report the results in the form of an Excel spreadsheet, formatted as in the following example:

Well ID	Donor	Stimulation	Stain (FITC/PE/PerCP-Cy5.5/APC)	%CD69+IFN□+
A01	105	none	anti-IFN□/CD69/CD4/CD3	0.02%
A02	105	none	anti-IFN□/CD69/CD8/CD3	0.05%
B01	105	SEB	anti-IFN□/CD69/CD4/CD3	14.3%
B02	105	SEB	anti-IFN□/CD69/CD8/CD3	20.6%
C01	105	pp65 pepmix	anti-IFN□/CD69/CD4/CD3	0.78%
C02	105	pp65 pepmix	anti-IFN□/CD69/CD8/CD3	1.04%
D01	105	SEB	IgG2a/IgG1/CD4/CD3	0.00%
D02	105	SEB	IgG2a/IgG1/CD8/CD3	0.01%

(note this spreadsheet can be automatically generated using Multiwell Plate Manager, by assigning the proper keywords to each sample and using batch analysis to generate the statistic of interest).
