# Supplementary material for: Standardization of cytokine flow cytometry assays
Source: BMC Immunol. 2005 Jun 24;6:13. doi: 10.1186/1471-2172-6-13 (PMC1184077; doi:10.1186/1471-2172-6-13)
Supplement: Additional File 2 — Protocol for shipped whole blood (Experiment 2) [file 1471-2172-6-13-S2.pdf]

CFC Standardization Protocol: Analysis of Shipped Whole Blood

Description

This protocol is for activation, processing, and analysis of shipped whole blood in 96-well deep well plates. The protocol is designed for four-color staining of IFN□-producing cells using a CD4 and a CD8 T cell staining cocktail.

Materials and Methods

Table 1 CFC Staining and Activation Reagents

CFC Reagents	Source	Catalog Number
BD FastImmune CD4 Intracellular IFN□ Detection Kit[b]	BDIS	Supplied
BD FastImmune CD8 Intracellular IFN□ Detection Kit	BDIS	Supplied
Deionized water[c]		
Paraformaldehyde, 10%	Electron Microscopy Sciences	15712-S
PBS 1X[c]		
Bovine serum albumin (BSA)[c]		
NaN_3[c]		
Staphylococcal enterotoxin B (SEB)	Sigma	Supplied
CMV pp65 peptide mix	BDIS	Supplied
DMSO	Sigma	D-8779
BD CaliBRITE™ FITC + PE beads	BDIS	349502
BD CaliBRITE PerCP-Cy5.5 beads	BDIS	345036
BD CaliBRITE APC beads	BDIS	340487

[a] BDIS: BD Biosciences, Immunocytometry Systems

[b] BD FastImmune intracellular detection kits include Brefeldin A, EDTA, BD FACS Lysing Solution, and BD FACS Permeabilization Solution 2.

[c] No specific manufacturer recommended

Table 2 Accessory Products and Instrumentation

Product	Source	Catalog Number
96-well deep well conical bottom plate	BDDL[a]	353966
Lid for 96-well deep well conical bottom plate	BDDL	351191
Single- and multi-channel pipettors and tips[b]		
Serological pipettor[b] (Pipet-Aid or equivalent) and pipets		
Table top centrifuge with deep well plate holders[b] (e.g. Sorvall RT6000 centrifuge, plate holder catalog #11093)		
37°C incubator or water bath[b]		
BD FACSCalibur brand flow cytometer	BDIS[a]	
BD Multiwell Autosampler (optional)	BDIS	342364
35 mm multiwell plate aspirator manifold	V&P Scientific, Inc. San Diego, CA 92121	VP 187A
Vacuum source for above[b]		

[a] BDIS: BD Biosciences, Immunocytometry Systems. BDDL: BD Biosciences, Discovery Labware

[b] No specific manufacturer recommended

Please follow all recommended precautions that are provided in the technical data sheet of each manufacturer's product.

Instructions for Processing Reagents

Antigens

SEB (positive activation control): Supplied as a working stock of 50 μg/ml in sterile PBS.

CMV pp65 peptide mix: On day of use, dilute 1:10 in sterile PBS to arrive at a working stock of 0.07 mg/mL per peptide.

Brefeldin A (BFA) from FastImmune kit

Upon receipt, thaw BFA, dispense into 30 μl aliquots, and store at -20ºC. On day of use, remove an aliquot from the freezer, and dilute 1:10 with sterile PBS. Discard any unused portion.

FACS Lysing Solution and FACS Permeabilizing Solution 2 from FastImmune kit

Dilute each 10X solution in deionized water to make 1X working solution. Store at room temperature.

Paraformaldehyde in PBS, 1%

Dilute 10% solution of paraformaldehyde 1:10 in 1X PBS. Store at 4ºC.

Wash buffer

First prepare stock solutions of 5% BSA in deionized water (filter sterilize) and 10% NaN_3 in deionized water. Then prepare 500 mL of wash buffer by adding 50 mL of 5% BSA stock solution and 5 mL of 10% NaN_3 stock solution to 445 mL of 1X sterile PBS. This represents final concentrations of 0.5% BSA and 0.1% NaN_3 in PBS. Store at 4ºC.

Protocol

Activation

1. Prepare working stock solution of pp65 peptide mix and 1:10 DMSO in PBS.
2. Label three tubes as "Control", "SEB," and "Peptide." Prepare stimulation reagents in bulk by combining stimulus (or 1:10 DMSO for control), BFA, and PBS into the appropriately labeled tubes. Amounts in the table below will provide more than enough reagent to stimulate blood from 3 donors, with two staining conditions for each stimulus (except SEB, for which two extra wells are plated for each donor and used for isotype control staining). If extra staining conditions are to be tested, the amounts below should be increased proportionally.

   Stimulus preparation for study:

Condition	Stimulus	BFA	PBS	Total volume
1:10 DMSO control	38 μL	30 μL	82 μL	150 μL
SEB	60 μL	60 μL	180 μL	300 μL
Pp65 peptide mix	38 μL	30 μL	82 μL	150 μL

3. Dispense 20 μl of appropriate stimulus reagent to each well of a 96-well deep well plate.
4. Add 200 μl heparinized whole blood to each well. Mix by gentle pipetting.
5. Incubate covered plate for six hours at 37°C.

Following incubation, cells may be held in covered plate at 18°C for up to 18 hours.

6. Add 20 □L of EDTA to each well and incubate for 15 minutes at room temperature. Mix each well by pipetting up and down.
7. Add 1.5 mL of 1X BD FACS Lysing Solution to each well. Incubate at room temperature for 10 minutes.
8. Centrifuge at room temperature at 500 x g for five minutes.
9. Aspirate supernatant. Pipet up and down in residual volume to loosen cell pellets.

> Cells may be frozen at this point: place covered plate, containing cells in residual FACS Lysing Solution, in -80°C freezer. When ready to stain, thaw plate at 37°C and continue as below.

Permeabilization and Staining

1. Add 1 mL of BD FACS Permeabilizing Solution 2 per well. Incubate at room temperature for 10 minutes.
2. Fill each well with 0.5 mL of wash buffer and centrifuge at room temperature at 500 x g for five minutes.
3. Aspirate supernatant.
4. Fill each well with 1.5 mL of wash buffer and centrifuge at room temperature at 500 x g for five minutes.
5. Aspirate supernatant.
6. Add appropriate staining mAbs to each well, pipetting to resuspend pellets. Incubate for 60 minutes at room temperature in the dark.
7. Fill each well with 1.5 mL of wash buffer and centrifuge at room temperature at 500 x g for five minutes.
8. Aspirate supernatant.
9. Repeat steps 7 and 8 one additional time.
10. Resuspend pellet with 200 □L cold 1% paraformaldehyde.
11. Keep plate at 4°C in the dark until FACS acquisition, which should be performed within 24 hours.

Acquisition

1. Using BD FACSComp™ software and BD CaliBRITE™ reagents, set voltages and compensation on flow cytometer (use Lyse No Wash settings in FACSComp). Be sure to replace the FL3 threshold created by FACSComp with an appropriate FSC threshold. Set an acquisition gate on lymphocytes.
2. Acquire 40,000 relevant (CD4+ or CD8+) events. Gating on CD3+CD4+ or CD3+CD8+ cells, report the % CD69+IFN□+ gated events for each sample. Also send the FCS files on a CD or Zip disk to: Holden Maecker, BD Biosciences, 2350 Qume Drive, San Jose, CA 95131. Thank you!
