# Supplementary material for: Standardization of cytokine flow cytometry assays
Source: BMC Immunol. 2005 Jun 24;6:13. doi: 10.1186/1471-2172-6-13 (PMC1184077; doi:10.1186/1471-2172-6-13)
Supplement: Additional File 3 — Protocol for cryopreserved PBMC (Experiment 3) [file 1471-2172-6-13-S3.pdf]

# CFC Standardization Protocol: Analysis of Cryopreserved PBMC

## Description

This protocol is for thawing, activation, processing, and analysis of cryopreserved PBMC in shallow 96-well round-bottom plates. The protocol is designed for four-color staining of IFNγ-producing cells using a CD4 and a CD8 T cell staining cocktail.

## Materials and Methods

### Table 1 CFC Staining and Activation Reagents

| CFC Reagents | Source | Catalog Number |
|---|---|---|
| BD FastImmune CD4 Intracellular IFNγ Detection Kit[b] | BDIS | Supplied |
| BD FastImmune CD8 Intracellular IFNγ Detection Kit | BDIS | Supplied |
| Deionized water[c] | | |
| Paraformaldehyde, 10% | Electron Microscopy Sciences | 15712-S |
| PBS 1X[c] | | |
| Bovine serum albumin (BSA)[c] | | |
| $NaN_3$[c] | | |
| Staphylococcal enterotoxin B (SEB) | Sigma | Supplied |
| CMV pp65 peptide mix | BDIS | Supplied |
| DMSO | Sigma | D-8779 |
| RPMI-1640, sterile, L-glutamine and HEPES supplemented | Sigma | R-7388 |
| Antibiotic/antimycotic solution | Sigma | A-9909 |
| Fetal bovine serum (FBS) | Sigma | F-2442 |
| BD CaliBRITE™ FITC + PE beads | BDIS | 349502 |
| BD CaliBRITE PerCP-Cy5.5 beads | BDIS | 345036 |
| BD CaliBRITE APC beads | BDIS | 340487 |

[a] BDIS: BD Biosciences, Immunocytometry Systems

[b] BD FastImmune intracellular detection kits include Brefeldin A, EDTA, BD FACS Lysing Solution, and BD FACS Permeabilization Solution 2.

[c] No specific manufacturer recommended

### Table 2 Accessory Products and Instrumentation

| Product | | Source | Catalog Number |
|---|---|---|---|
| 50 mL polypropylene conical tube | | BDDL[a] | 352070 |
| 96-well round bottom plate with lid | | BDDL | 353077 |
| Disposable polystyrene serological pipette: | 5 mL | BDDL | 357543 |
| | 10 mL | | 357551 |
| Trypan blue, 0.4% solution | | Sigma | T-8154 |
| Hemacytometer[b] | | | |
| Ice bucket[b] | | | |

| | | |
|---|---|---|
| Single- and multi-channel pipettors and tips [b] | | |
| Serological pipettor[b] (Pipet-Aid or equivalent) | | |
| Light microscope[b] | | |
| Table top centrifuge with tube buckets and plate holders[b] | | |
| 37°C incubator or water bath[b] | | |
| BD FACSCalibur brand flow cytometer | BDIS[a] | |
| BD Multiwell Autosampler | BDIS | 342364 |
| 7 mm multiwell plate aspirator manifold | V&P Scientific, Inc. San Diego, CA 92121 | Custom order |
| Vacuum source for above[b] | | |

[a] BDIS: BD Biosciences, Immunocytometry Systems. BDDL: BD Biosciences, Discovery Labware

[b] No specific manufacturer recommended

Please follow all recommended precautions that are provided in the technical data sheet of each manufacturer's product.

### Instructions for Processing Reagents

#### Antigens

SEB (positive activation control): Add 2 mL of sterile PBS directly to a 1-mg vial of SEB. Cap the vial and shake to dissolve all the powder. Store this stock solution at 4ºC. On day of use, prepare working stock by diluting 1:10 in sterile PBS.

CMV pp65 peptide mix: On day of use, dilute 1:10 in sterile PBS to arrive at a working stock of 0.07 mg/mL per peptide.

#### Brefeldin A (BFA) from FastImmune kit

Upon receipt, thaw BFA, dispense into 30 μl aliquots, and store at $^{-}$20ºC. On day of use, remove an aliquot from the freezer, and dilute 1:10 with sterile PBS. Discard any unused portion.

#### FACS Lysing Solution and FACS Permeabilizing Solution 2 from FastImmune kit

Dilute each 10X solution in deionized water to make 1X working solution. Store at room temperature.

#### Paraformaldehyde in PBS, 1%

Dilute 10% solution of paraformaldehyde 1:10 in 1X PBS. Store at 4ºC.

#### Wash buffer

First prepare stock solutions of 5% BSA in deionized water (filter sterilize) and 10% $NaN_3$ in deionized water. Then prepare 500 mL of wash buffer by adding 50 mL of 5% BSA stock solution and 5 mL of 10% $NaN_3$ stock solution to 445 mL of 1X sterile PBS. This represents final concentrations of 0.5% BSA and 0.1% $NaN_3$ in PBS. Store at 4ºC.

#### Complete RPMI (cRPMI)

Supplement sterile RPMI-1640 medium with 10% sterile heat-inactivated FBS and 1% sterile antibiotic/antimycotic. Store at 4ºC.

## Protocol

### Preparation

1. Place cRPMI media at 37°C in a water bath for about 30 minutes.
2. Label a 50 ml centrifuge tube per sample and add 8 mls of warm (22°C to 37°C) assay media.
3. Remove cryovials from liquid nitrogen freezer and place directly on dry ice.

4. Thaw no more than 2 cryovials at a time. Place cryovials in a 37°C water bath until cell suspension is almost completely melted or a small bit of ice remains.
5. Dry off the outside of the cryovials and wipe with 70% ethanol.
6. Add 1 ml of warm (22°C to 37°C) cell culture media to the thawed cells slowly.
7. Transfer the cell suspension to the 50 ml polypropylene tubes containing 8 mls of media.
8. Balance tubes and centrifuge at 1200 rpm for 10 minutes.
9. Aspirate supernatant and resuspend cells in 2 ml assay medium.
10. Perform manual cell count using Trypan Blue to determine PBMC viability.
11. Resuspend at a final concentration of $5x10^6$ live PBMCs/mL in warm cRPMI media.
12. Allow any visible clumps to settle to the bottom and avoid pipetting them.
13. Plate 200 µl/well in a round-bottom 96-well plate. This will result in $1x10^6$ cells per well (we have tested $5x10^5$ to $2x10^6$ cells per well with equivalent results). Prepare at least eight wells for each thawed PBMC specimen (two for DMSO control, four for SEB stimulation, and two for pp65 peptide mix stimulation). Prepare additional wells for manual compensation, if desired.
14. Incubate covered plate at 37°C for 12–18 hours.

## Activation

1. Prepare working stock solutions of activating reagents.
2. Label three tubes as "Control", "SEB," and "Peptide." Prepare stimulation reagents in bulk by combining stimulus (or DMSO for control), BFA, and PBS into the appropriately labeled tubes. Amounts in the table below will provide more than enough reagent to stimulate blood from 6 donors, with two staining conditions for each stimulus (except SEB, for which two extra wells are plated for each donor and used for isotype control staining). If extra staining conditions are to be tested, the amounts below should be increased proportionally.

   Stimulus preparation for study:

| Condition | Stimulus | BFA | PBS | Total volume |
|---|---|---|---|---|
| DMSO control | 60 µL DMSO | 60 µL | 180 µL | 300 µL |
| SEB | 120 µL | 120 µL | 360 µL | 600 µL |
| Peptide Mix | 75 µL | 60 µL | 165 µL | 300 µL |

3. Dispense 20 µl of appropriate stimulus reagent to each well of the plate containing PBMC, mixing by pipetting up and down.
4. Incubate covered plate for six hours at 37°C.

> Following incubation, cells may be held in covered plate at 18°C for up to 18 hours.

5. Add 20 µL of EDTA to each well and incubate for 15 minutes at room temperature. Mix well by pipetting up and down.
6. Centrifuge plate at 250 x g for five minutes; aspirate supernatant with manifold. Approximately 30 µl will remain in wells.
7. Resuspend each well with 100 µL of 1X BD FACS Lysing Solution. Incubate at room temperature for 10 minutes.

> Cells may be frozen at this point: place covered plate, containing cells in FACS Lysing Solution, in -80°C freezer. When ready to stain, thaw plate at 37°C and continue as below.

8. Add 100 µL of wash buffer to each well and centrifuge at room temperature at 500 x g for five minutes.

9. Aspirate supernatant with manifold.

### Permeabilization and Staining

1. Aspirate supernatant and resuspend cells in 200 µL of BD FACS Permeabilizing Solution 2 per well. Incubate at room temperature for 10 minutes.
2. Centrifuge at room temperature at 500 x g for five minutes.
3. Aspirate supernatant with manifold.
4. Resuspend cells in 200 µL of wash buffer per well and centrifuge at room temperature at 500 x g for five minutes.
5. Aspirate supernatant with manifold.
6. Repeat steps 4 and 5 a second time to remove all traces of Permeabilizing Solution prior to staining.
7. Add appropriate staining mAbs to each well, pipetting up and down to mix. For each specimen and stimulus, add 20 µL of CD4 cocktail to one well, and 20 µL of CD8 cocktail to the second well. For SEB stimulations, also add 20 µL of CD4 isotype control cocktail to the third well, and 20 µL of CD8 isotype control cocktail to the fourth well. Incubate for 30-60 minutes at room temperature in the dark.
8. Add 200 µL of wash buffer to each well and centrifuge at room temperature at 500 x g for five minutes.
9. Aspirate supernatant with manifold.
10. Repeat steps 8 and 9 a second time.
11. Resuspend pellet with 200 µL cold 1% paraformaldehyde.
12. Keep plate at 4°C in the dark until FACS acquisition, which should be performed within 2-18 hours.

### Acquisition

1. Using BD FACSComp™ software and BD CaliBRITE™ reagents, set compensation on BD FACSCalibur using Lyse No Wash settings. Be sure to remove the FL3 threshold that is automatically set, and replace it with an appropriate FSC threshold. Set an acquisition gate on lymphocytes.
2. Acquire at least 10,000 relevant (CD4+ or CD8+) events, preferably 40,000. Report the %CD69+IFNg+CD4+ and %CD69+IFNg+CD8+ cells for each specimen in an Excel spreadsheet, and also send the original FCS files on a Mac Zip disk or CD to:

Holden Maecker
BD Biosciences
2350 Qume Drive
San Jose, CA 95131
