# Supplementary material for: Standardization of cytokine flow cytometry assays
Source: BMC Immunol. 2005 Jun 24;6:13. doi: 10.1186/1471-2172-6-13 (PMC1184077; doi:10.1186/1471-2172-6-13)
Supplement: Additional File 4 — Protocol for cryopreserved PBMC with lyophilised antigen and antibody plates (Experiment 4) [file 1471-2172-6-13-S4.pdf]

# CFC Standardization Protocol: Analysis of Cryopreserved PBMC Using Lyophilized Reagent Plates

## Description

This protocol is for the thawing, activation, processing, and analysis of cryopreserved PBMC in shallow 96-well V-bottom plates, using activation and staining reagents that have been lyophilized in these plates. The protocol is designed for four-color staining of cytokine-producing cells using three lyophilized antibody cocktails (provided) as well as another liquid antibody cocktail of the user's choice.

## Materials

### Table. Reagents and Accessories

| Product | Suggested Source | Catalog Number |
|---|---|---|
| Activation plate | BD Biosciences | Supplied |
| Staining plate | BD Biosciences | Supplied |
| EDTA, 20 mM solution | BD Biosciences | Supplied |
| BD FACS Lysing Solution | BD Biosciences | Supplied |
| BD FACS Permeabilizing Solution 2 | BD Biosciences | Supplied |
| BD CaliBRITE™ FITC + PE beads[1] | BD Biosciences | 349502 |
| BD CaliBRITE PerCP-Cy5.5 beads[1] | BD Biosciences | 345036 |
| BD CaliBRITE APC beads[1] | BD Biosciences | 340487 |
| Paraformaldehyde, 10% | Electron Microscopy Sciences | 15712-S |
| RPMI-1640, sterile, L-glutamine and HEPES supplemented | Sigma | R-7388 |
| Antibiotic/antimycotic solution | Sigma | A-9909 |
| Fetal bovine serum (FBS) | Sigma | F-2442 |
| Trypan blue, 0.4% solution | Sigma | T-8154 |
| Deionized water | Any source | |
| PBS 1X | Any source | |
| Bovine serum albumin (BSA) | Any source | |
| $NaN_3$ | Any source | |
| 50 mL polypropylene conical tube | Any source | |
| Disposable polystyrene serological pipette: 5 mL, 10 mL | Any source | |
| Hemacytometer | Any source | |
| Ice bucket | Any source | |
| Single- and multi-channel pipettors and tips | Any source | |
| Serological pipettor (Pipet-Aid or equivalent) | Any source | |
| Light microscope | Any source | |
| Table top centrifuge with tube buckets and plate holders | Any source | |
| 37°C incubator or water bath | Any source | |
| 7 mm multiwell plate aspirator manifold | V&P Scientific, Inc. http://www.vp-scientific.com/ | VP187D |
| Vacuum source for aspirator manifold | Any source | |

[1]optional, for instrument set-up

### Required Solutions

FACS Lysing Solution and FACS Permeabilizing Solution 2
Dilute each 10X solution in deionized water to make 1X working solution. Store at room temperature.

1% Paraformaldehyde in PBS
Dilute 10% solution of paraformaldehyde 1:10 in 1X PBS. Store at 4ºC.

Wash Buffer
Prepare 0.5% BSA and 0.1% $NaN_3$ in PBS. Store at 4ºC.

Complete RPMI (cRPMI)
Supplement sterile RPMI-1640 medium with 10% sterile heat-inactivated FBS and 1% sterile antibiotic/antimycotic. Store at 4ºC.

## Protocol

### Thawing and Resting of PBMC

1. Place cRPMI media in a 37°C water bath for about 30 minutes.
2. Work in a tissue culture hood for steps 2-9. Label a 50 mL centrifuge tube for each sample and add 8 mL of warm (37°C) cRPMI media.
3. Remove cryovials from liquid nitrogen freezer and place directly on dry ice.
4. Thaw no more than 2 cryovials at a time. Place cryovials in a 37°C water bath until cell suspension is almost completely melted or a small bit of ice remains.
5. Dry off the outside of the cryovials and wipe with 70% ethanol.
6. Add 1 mL of warm (37°C) cRPMI media to the thawed cells slowly.
7. Transfer the cell suspension to the 50 mL polypropylene tube(s) containing 8 mL of media. If duplicate cryovials from the same donor are being thawed, the cells can be added to a single 50 mL tube with 16 mL of media.
8. Balance tubes and centrifuge at 250 x g for 10 minutes.
9. Aspirate supernatant and resuspend cells in 5 mL cRPMI medium.
10. Loosen the tube cap, and lay it onto a 5- or 10-mL pipet in a 37°C $CO_2$ incubator, so that the tube rests at a slant but the media does not reach the top of the tube. Incubate overnight (12-18 hours).

### Activation and Fixation

11. Re-warm cRPMI media in 37°C water bath. Vortex cells to resuspend. Perform a cell count using trypan blue to determine PBMC concentration and viability.
12. Centrifuge at 250 x g for 5 minutes, and resuspend at a final concentration of $5x10^6$ live PBMC/mL in warm cRPMI media.
13. Allow any visible clumps to settle to the bottom, and avoid pipetting them.
14. Open the pouch marked “Lyophilized Antigen Plate” (red lettering). Tap the sealed plate on the bench until all pellets are resting in the bottom of the wells, then carefully peel off the plate seal. Keep the red label on the plate facing you to keep the plate oriented with well A1 at the top left.
15. Add 200 □l/well of cell suspension to the appropriate wells of the plate (see map below). This will result in $1x10^6$ cells per well. After all cells have been dispensed as desired, pipette the entire volume of each well up and down 10 times to thoroughly dissolve all antigen pellets.

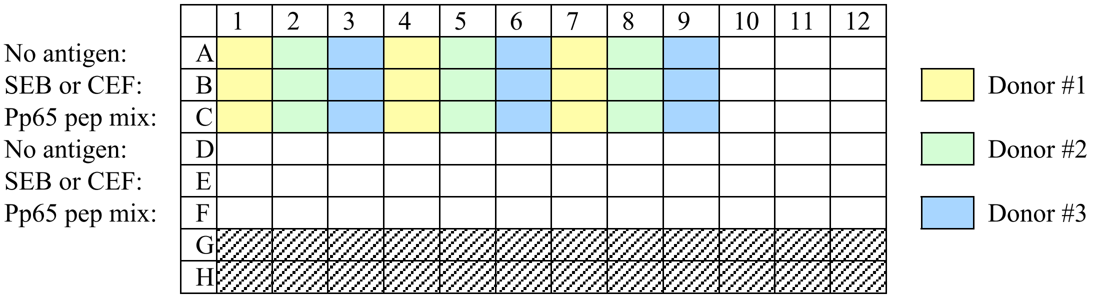


Figure 1. Cell filling pattern for Lyophilized Antigen Plate for single replicates. For duplicate wells, use rows D-F if desired and if cell numbers allow. For testing of liquid antibodies, use columns 10-12 if desired. Do not add cells to rows G-H, as these will contain lyophilized control cells.

16. Cover plate and incubate at 37°C for 6 hours.

> Following incubation, cells may be held in covered plate at 4-18°C for up to 18 hours.

17. Add 20 µL of EDTA to each well, mix well by pipetting up and down, and incubate for 15 minutes at room temperature.
18. Centrifuge plate at room temperature at 250 x g for five minutes. Aspirate supernatant with manifold.
19. Resuspend the cells in each well with 100 µL of 1X BD FACS Lysing Solution. Incubate at room temperature for 10 minutes.

> Cells may be frozen at this point: place covered plate, wrapped in parafilm and containing cells in FACS Lysing Solution, in -80°C freezer. When ready to stain, place plate at 37°C just until thawed, and continue as below.

20. Add 100 µL of wash buffer to each well and centrifuge at room temperature at 500 x g for five minutes. Aspirate supernatant with manifold.

## Permeabilization and Staining

21. Resuspend cells in 200 µL of BD FACS Permeabilizing Solution 2 per well. Incubate at room temperature for 10 minutes.
22. Centrifuge plate at room temperature at 500 x g for five minutes. Aspirate supernatant with manifold.
23. Resuspend cells in 200 µL of wash buffer per well and centrifuge plate at room temperature at 500 x g for five minutes. Aspirate supernatant with manifold.
24. Repeat step 23 a second time to remove all traces of Permeabilizing Solution prior to staining.
25. Open the pouch marked "Lyophilized Antibody Plate" (blue lettering). Tap the sealed plate on the bench and ensure that all pellets are resting in the bottom of the wells, then peel off the plate seal. Keep the blue label on the plate facing you to keep the plate oriented with well A1 at the top left.
26. Add 55 µL of wash buffer to each well of the Lyophilized Antibody Plate. Be sure to include the wells in rows G-H that include lyophilized control cells. After liquid has been dispensed to all wells, pipette the entire well volume up and down in each well 10 times. Transfer 50 µL from each well of the antibody plate to each well of the cell plate (don't forget to transfer control cell wells). Add liquid antibody to appropriate wells in columns 10-12 of the cell plate, if applicable. Pipette up and down to mix.
27. Incubate for 60 minutes at room temperature in the dark.
28. Add 200 µL of wash buffer to each well and centrifuge plate at room temperature at 500 x g for five minutes. Aspirate supernatant with manifold.
29. Repeat step 28 a second time.
30. Resuspend pellet with 200 µL cold 1% paraformaldehyde in PBS.
31. Keep plate covered at 4°C in the dark until acquisition, which should be performed within 24 hours.

## Acquisition and Analysis

32. (A) Automated setup: Using BD FACSComp™ software and BD CaliBRITE™ reagents, set compensation on BD FACSCalibur using Lyse No Wash settings. Be sure to remove the FL3 threshold that is automatically set, and replace it with an appropriate FSC threshold. Adjust FSC/SSC gains if necessary.
    (B) Manual setup: Alternatively, use negative control cells (well G10 or H10) to set PMT and FSC gain values so that the lymphocytes span the first decade of each fluorescence parameter and can be clearly resolved from monocytes and debris in FSC and SSC parameters. Set an appropriate threshold on FSC to eliminate debris. Then set compensation using the single-stained control cells (wells G11, G12, H11, H12).
33. Samples can be acquired using either an automated plate loader or by transferring the cells to tubes if no loader is available. Set an acquisition gate on lymphocytes (in a FSC vs. SSC plot) and on cells positive for CD3 (in a CD3 versus SSC plot). For each sample, acquire 60,000 CD3+ events, if possible. Store all events. Be sure to annotate the files with the appropriate parameters as in the spreadsheet below, i.e., please enter the donor ID and stimulus into either MPM, HTS, or CellQuest software, using keywords or sample ID/patient ID fields. Label the parameters with the staining antibody conjugates for each sample. Please name the files by site (BD, Duke, IAVI, etc.), experiment (LYO1), and well ID as in the example below. If you are not using a plate loader, your file names will not automatically be given well ID suffixes; in this case please manually add the well ID at the end of the file name.
34. Calculate the %cytokine+ cells among CD3+CD4+ and CD3+CD8+ cells for the first antibody cocktail. Calculate the %CD69+IFN[illegible]+ cells among CD3+CD4+ or CD3+CD8+ cells for the second and third antibody cocktail, respectively. Report the results for each sample in an Excel spreadsheet (see example below, automatically generated from MPM with minor modifications). Include analysis of the three prestained control cells (wells H1, H4, and H7). Do not include any analysis of the negative control or single-stained control cells. E-mail this spreadsheet, along with a list of the viability and recovery obtained for each donor, to:

    holden_maecker@bd.com

    Also send the original FCS files on a Mac Zip disk or CD (no DVD's please) to:

    Holden Maecker
    BD Biosciences
    2350 Qume Drive
    San Jose, CA 95131 USA

| Well | Donor | Stimulus | Data File Name | FL1 | FL2 | FL3 | FL4 | CD4 cyt+ | (bkgd subt) | CD8 cyt+ | (bkgd subt) |
|---|---|---|---|---|---|---|---|---|---|---|---|
| A01 | 1 | unstim | BDLYO1.A01 | CD4 FITC | IFNg+IL-2 PE | CD8 PerCP-Cy5.5 | CD3 APC | 0.04 | | 0.02 | |
| A02 | 1 | SEB | BDLYO1.B01 | CD4 FITC | IFNg+IL-2 PE | CD8 PerCP-Cy5.5 | CD3 APC | 2.71 | 2.67 | 5.64 | 5.62 |
| A03 | 1 | pp65 | BDLYO1.C01 | CD4 FITC | IFNg+IL-2 PE | CD8 PerCP-Cy5.5 | CD3 APC | 0.17 | 0.13 | 4.03 | 4.01 |
| A04 | 2 | unstim | BDLYO1.A02 | CD4 FITC | IFNg+IL-2 PE | CD8 PerCP-Cy5.5 | CD3 APC | 0.08 | | 0.12 | |
| A05 | 2 | SEB | BDLYO1.B02 | CD4 FITC | IFNg+IL-2 PE | CD8 PerCP-Cy5.5 | CD3 APC | 10.93 | 10.85 | 14.60 | 14.48 |
| A06 | 2 | pp65 | BDLYO1.C02 | CD4 FITC | IFNg+IL-2 PE | CD8 PerCP-Cy5.5 | CD3 APC | 0.55 | 0.47 | 0.23 | 0.11 |
| A07 | 3 | unstim | BDLYO1.A03 | CD4 FITC | IFNg+IL-2 PE | CD8 PerCP-Cy5.5 | CD3 APC | 0.01 | | 0.03 | |
| A08 | 3 | SEB | BDLYO1.B03 | CD4 FITC | IFNg+IL-2 PE | CD8 PerCP-Cy5.5 | CD3 APC | 5.58 | 5.57 | 8.10 | 8.07 |
| A09 | 3 | pp65 | BDLYO1.C03 | CD4 FITC | IFNg+IL-2 PE | CD8 PerCP-Cy5.5 | CD3 APC | 1.34 | 1.33 | 0.55 | 0.52 |
| … | … | … | … | … | … | … | … | … | … | … | … |

Figure 2. Example of reporting spreadsheet format and appropriate file annotation (donor, stimulus, etc.). Please follow this format precisely to allow easier, error-free data compilation! Thanks!
